# The Response of *COL* and *FT* Homologues to Photoperiodic Regulation in Carrot (*Daucus carota* L.)

**DOI:** 10.1038/s41598-020-66807-y

**Published:** 2020-06-19

**Authors:** Lijie Liu, Chenggang Ou, Shumin Chen, Qi Shen, Bo Liu, Min Li, Zhiwei Zhao, Xiaoping Kong, Xiangping Yan, Feiyun Zhuang

**Affiliations:** 1 Key Laboratory of Horticultural Crop Biology and Germplasm Innovation, Ministry of Agriculture; Institute of Vegetables and Flowers, Chinese Academy of Agricultural Science No. 12 Nanda Street, Zhongguan Cun, Haidian District, Beijing, 100081 China; 2 College of Ecological Environment and Resources, Qinghai Nationalities University No. 3, Bayi Middle Road, Chengdong District, Xining, Qinghai Province 810007 China; 3 Xining Institute of Vegetables, Xining No. 4 Weisan Road, Biological Industry Park, Xining, 810016 Qinghai China

**Keywords:** Genetics, Gene expression, Flowering, Plant genetics, Plant evolution

## Abstract

Carrot (*Daucus carota* L.) is a biennial plant requiring vernalization to induce flowering, but long days can promote its premature bolting and flowering. The basic genetic network controlling the flowering time has been constructed for carrot, but there is limited information on the molecular mechanisms underlying the photoperiodic flowering response. The published carrot genome could provide an effective tool for systematically retrieving the key integrator genes of *GIGANTEA* (*GI*), *CONSTANS-LIKE* (*COL*), *FLOWERING LOCUS T* (*FT*), and *SUPPRESSOR OF OVEREXPRESSION OF CONSTANS 1* (*SOC1*) homologues in the photoperiod pathway. In this study, the bolting time of wild species “Songzi” (Ws) could be regulated by different photoperiods, but the orange cultivar “Amsterdam forcing” (Af) displayed no bolting phenomenon. According to the carrot genome and previous de novo transcriptome, 1 *DcGI*, 15 *DcCOL*s, 2 *DcFT*s, and 3 *DcSOC1*s were identified in the photoperiod pathway. The circadian rhythm peaks of *DcGI*, *DcCOL*2, *DcCOL5a*, and *DcCOL13b* could be delayed under long days (LDs). The peak value of *DcCOL2* in Af (12.9) was significantly higher than that in Ws (6.8) under short day (SD) conditions, and was reduced under LD conditions (5.0). The peak values of *DcCOL5a* in Ws were constantly higher than those in Af under the photoperiod treatments. The expression levels of *DcFT1* in Ws (463.0) were significantly upregulated under LD conditions compared with those in Af (1.4). These responses of *DcCOL2*, *DcCOL5a*, and *DcFT1* might be related to the different bolting responses of Ws and Af. This study could provide valuable insights into understanding the key integrator genes in the carrot photoperiod pathway.

## Introduction

The floral transition from a vegetative to a reproductive state is a critically important stage in the lifecycle of plants. Vernalization, photoperiod, ambient temperature, autonomous, gibberellin, and age pathways comprise a sophisticated regulatory network containing multiple endogenous and external factors that control flowering in *Arabidopsis*^1,2^. These pathways have their own unique initiations, but eventually converge to downstream key floral integrator genes, such as *FLOWERING LOCUS T* (*FT*) and *SUPPRESSOR OF OVEREXPRESSION OF CONSTANS 1* (*SOC1*)^1–3^. The FT protein, as a mobile florigen, moves from leaves to the shoot apical meristem (SAM) and interacts with FLOWERING LOCUS D (FD) to form a complex, which upregulates SOC1 to induce the meristem identity genes to reprogram the primordia to form reproductive organs^4–7^. SOC1 can interact with the MADS-box transcription factor AGAMOUS-LIKE 24 (ALG24) to provide a positive feedback loop and activate *LEAFY (LFY)* expression by directly binding to its promoter^8–10^. Through the effect of these integrator genes, the floral meristem-determining genes are consequently activated to control the flowering time^8,11^. This progress in the model plant *Arabidopsis* provides an important reference for researching floral molecular mechanisms in other crops^11,12^. In fact, there are usually more than two or three interconnected pathways involved in regulating flowering under natural conditions^3,12–14^.

In the photoperiod pathway, the functional hierarchy *GIGANTEA* (*GI*)–*CONSTANS* (*CO*)–*FT* has been identified, and plays a key role in regulating flowering^7,11,15–18^. During the late afternoons of long days (LDs), GI forms a complex with the FLAVIN-BINDING, KELCH REPEAT, F BOX 1 (FKF1) to degrade CYCLING DOF FACTORs (CDFs), which contains the major regulators of *CO* transcription, and consequently, CO protein accumulates to promote flowering by activating the transcription of *FT* and *TWIN SISTER OF FT* (*TSF*) in the leaf vasculature^5,7,8,19–21^. The degradation complex cannot be formed during short days (SDs) due to *GI* and *FKF1* expression not coinciding^20,22^. In rice, *HEADING DATE 1* (*Hd1*), which is the homologue of *CO*, promotes flowering by activating the expression of the *FT*-like gene *HEADING DATE 3a* (*Hd3a*) on SDs, and represses flowering on LDs^12,15,23,24^. By integrating light and circadian clock signals to regulate the downstream florigen gene, *CO* plays a central role in the mechanism of photoperiod flowering in *Arabidopsis*^3,11,18^. Extensive gene duplication events have occurred in this gene family, resulting in 16 other *CO*-like (*COL*) genes with different functions^25,26^. Among them, *COL1* and *COL2* have little effect on the flowering time, while *COL3*, *COL4*, and *COL9* represent flowering repressors^27–29^. The overexpression of *COL8* delays flowering under LDs, whereas *COL5* promotes flowering^30,31^. Multiple *CO/COL* homologues have been identified in different species: 16 in rice, 9 in barley, 13 in sugar beet, and 28 in soybean^32–35^. *GmCOL5* can rescue the late-flowering phenotype of the *co* mutant^33^. *OsCOL13* negatively regulates flowering under LD and SD conditions in rice^36^. There is little evidence to prove that *CO* homologues are potential regulators of *FT*-like genes^12^.

The flowering habits of many domesticated crops have changed greatly following human selection, which provides an important route for studying flowering mechanisms by comparing cultivars with their ancestors and wild relatives^37–40^. Carrot (*Daucus carota* L.) is a biennial plant requiring vernalization to induce flowering, and the seedling is usually not responsive to a low temperature (between 0 and 10 °C) until it has 8–12 leaves^41^. Moreover, long days can promote premature bolting in carrot^42^. With a change of cultivation system, carrots can be harvested and abundantly supplied all year round in markets. The premature bolting of carrot has been a serious risk during the winter–spring period or spring cultivation. The seedlings grow under a low temperature for a long time at the early stage and grow under long days at the late stage, this is why some varieties which are not tolerant to bolting are prone to bolting. Selection tolerance to premature bolting has been a constant concern in carrot breeding^43–46^. The wild species *D. carota* subsp. *carota* Songzi (Ws) is sensitive to flower induction by vernalization and photoperiod, and orange cultivar *D. carota* var. *sativus* Amsterdam forcing (Af) is tolerant^42^. Through a de novo transcriptome comparison of Ws and Af, a basic genetic network controlling the flowering time was constructed, including photoperiod, vernalization, and gibberellin pathways^42^. Furthermore, an *FT*-like gene was identified in carrot^47^. There is substantial evidence that *FT* homologues have a conserved role in promoting flowering and reflect variation in the copy number in divergent angiosperms^12^. Despite carrot being one of the 10 most important vegetables, understanding on the molecular mechanisms of *COL* and *FT* homologues underlying the photoperiodic flowering response is limited. In this study, new homologues of *COL*, *FT*, and *SOC1* were retrieved based on the carrot genome^48^. The structure, phylogenetic relationship, and molecular evolutionary rate variation of *GI*, *COL*, *FT*, and *SOC1* homologues were investigated using 21 *D. carota* var. *sativus* accessions (as DCS), 4 *D. carota* subsp. *gummifer* species and 9 *D. carota* subsp. *carota* species (as DCC), and 5 *Daucus* species (as Dau) (Supplementary Table S1). The circadian rhythms of *GI*, *COL*, *FT*, and *SOC1* homologues under different photoperiods and their expression trends during growth in different seasons were analyzed in Ws and Af. *DcCOL2* and *DcCOL5a* were transformed into *Arabidopsis thaliana* plants to analyze their function in regulating flowering. This study provides an improved understanding of the regulatory network of photoperiodic flowering in carrots.

## Results

### Phylogenetic and nucleotide diversity of *DcGI*, *DcCOL*s, *DcFT*s, and *DcSOC1*s

**Figure 1:**
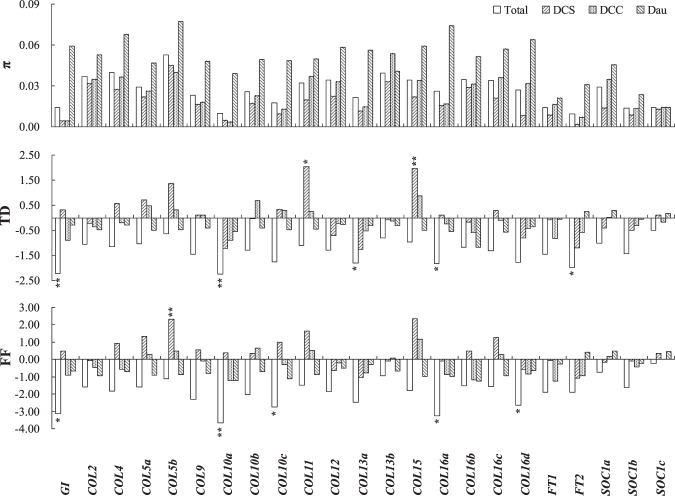
Nucleotide diversity (π) and neutrality tests of *DcGI*, *DcCOL*s, *DcFT*s, and *DcSOC1*s. DCS presents 21 *Daucus carota* var. *sativus* accessions, DCC presents 4 *D. carota* subsp. *gummifer* species and 9 *D. carota* subsp. *carota* species, Dau presents 5 *Daucus* species, and Total presents all 39 accessions. Neutral tests of Tajima’s D (TD) and Fu and Li’s F (FF) were estimated based on the neutral model prediction by DnaSP 6. * and ** represent a 0.05 and 0.01 significance level.

**Figure 2:**
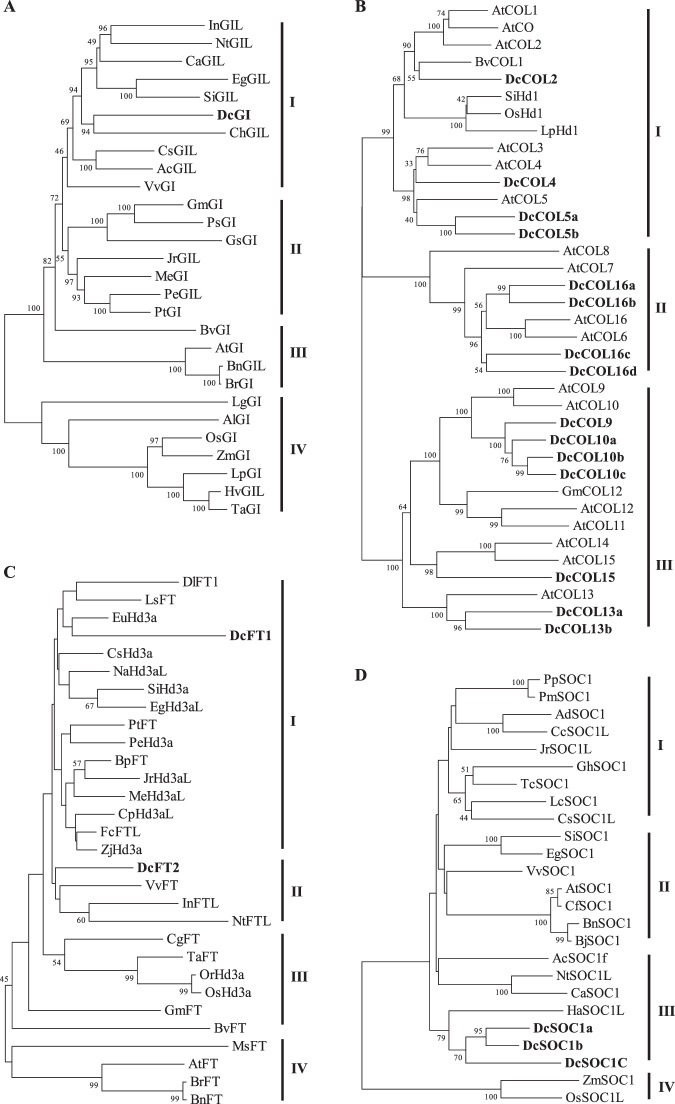
Phylogenetic analysis of *GIGANTEA* (*GI*), *CONSTANS-LIKE* (*COL*), *FLOWERING LOCUS T* (*FT*), and *SUPPRESSOR OF OVEREXPRESSION OF CONSTANS 1* (*SOC1*) protein sequences in different plants. Phylogenetic analysis was performed using the neighbor-joining method by MEGA5.0. **(A)** The phylogenetic tree of *GI*/*GI-like* protein sequences was divided into three groups. Ac, *Actinidia chinensis* PSS17443.1; Al, *Allium cepa* ACT22764.1; At, *Arabidopsis thaliana* AAF00092.1; Bn, *Brassica napus* XP013725974.1; Br, *Brassica rapa* NP001288824.1; Bv, *Beta vulgaris* XP010681268.1; Ca, *Coffea arabica* XP027093176.1; Ch, *Chrysanthemum seticuspe* BAM67030.1; Cs, *Camellia sinensis* XP028106519.1; Dc, *D. carota* XP017226526.1; Eg, *Erythranthe guttata* XP012838945.1; Gm, *Glycine max* BAJ22595.1; Gs, *Glycine soja* KHN41309.1; Hv, *Hordeum vulgare* AAW66945.1; In, *Ipomoea nil* XP019154767.1; Jr. *Juglans regia* XP018846578.1; Lg, *Lemna gibba* BAD97869.1; Lp, *Lolium perenne* CAY26028.1; Me, *Manihot esculenta* XP021601157.1; Nt, *Nicotiana tabacum* XP016470513.1; Os, *Oryza sativa* XP015649578.1; Pe, *Populus euphratica* XP011042897.1; Ps, *Pisum sativum* ABP81863.1; Pt, *Populus trichocarpa* XP002307516.2; Si, *Sesamum indicum* XP011074103.1; Ta, *Triticum aestivum* AAQ11738.1; Vv, *Vitis vinifera* XP002264755.1; Zm, *Zea mays* ABZ81992.1. (**B**) The phylogenetic tree of *CO*/*COL* protein sequences was divided into four groups. At, *A. thaliana* AED92213.1 (CO), NP197089.1 (COL1), NP186887 (COL2), NP180052 (COL3), NP197875.2 (COL4), NP568863 (COL5), NP564932 (COL6), NP177528 (COL7), NP001031160 (COL8), NP001118599 (COL9), NP199636 (COL10), NP193260.2 (COL11), NP188826 (COL12), O82256 (COL13), NP850211 (COL14), Q9C7E8 (COL15), and NP173915 (COL16); Os, *Oryza sativa* NP001057378; Gm, *Glycine max* XP014619827.1; Lp, *Lolium perenne* CAM31943.1; Bv, *Beta vulgaris* ACC95129; Si, *Setaria italica* BAN00014.1; Dc, *D. carota* XP017231361.1 (DcCOL2), XP017230434.1 (DcCOL4), XP017237885.1 (DcCOL5a), XP017255010.1 (DcCOL5b), XP017238354.1 (DcCOL9), XP017243761.1 (DcCOL10a), XP017253919.1 (DcCOL10b), XP017215116.1 (DcCOL10c), XP017229749.1 (DcCOL13a), XP017243283.1 (DcCOL13b), XP017219330.1 (DcCOL15), XP017237500.1 (DcCOL16a), XP017244294.1 (DcCOL16b), XP017219277.1 (DcCOL16c), and XP017223731.1 (DcCOL16d). **(C)** The phylogenetic tree of *FT*/*Hd3a* protein sequences was divided into four groups. At, *A. thaliana* BAA77838.1; Bn, *Brassica napus* XP013699257.1; Bp, *Betula platyphylla* AFR31531.1; Br, *Brassica rapa* XP009127403.1; Bv, *Beta vulgaris* XP010690385.1; Cg, *Cymbidium goeringii* ADI58462.1; Cp. *Carica papaya* XP021911503.1; Cs, *Camellia sinensis* XP028086172.1; Dc, *D. carota* XP017225396.1 (DcFT1) and XP017216959.1 (DcFT2); Dl, *Dimocarpus longan* AEZ63949.1; Eg, *Erythranthe guttata* XP012834843.1; Eu, *Eucalyptus grandis* XP010038562.1; Fc, *Fagus crenata* BAP28173.1; Gm, *Glycine max* NP001240185.1; In, *Ipomoea nil* ABW73563.1; Jr, *Juglans regia* XP018856683.1; Ls, *Lactuca sativa* BAK14368.1; Me, *Manihot esculenta* XP021633631.1; Ms, *Medicago sativa* AEO16612.1; Na, *Nicotiana attenuata* XP019265970.1; Nt, *Nicotiana tabacum* XP016507270.1; Or, *Oryza rufipogon* BAO03055.1; Os, *Oryza sativa* XP015641951.1; Pe, *Populus euphratica* XP011008885.1; Pt, *Populus trichocarpa* XP002316173.1; Si, *Sesamum indicum* XP011084685.1; Ta, *Triticum aestivum* AAW23034.1; Vv, *Vitis vinifera* NP001267907.1; Zj, *Ziziphus jujuba* XP015873598.1. (**D)** The phylogenetic tree of *SOC1* protein sequences were divided into four groups. Ac, *Actinidia chinensis* AKH61959.1; Ad, *Arachis duranensis* XP015961558.1; At, *A. thaliana* AEC10583.1; Bj, *Brassica juncea* AFH41827.1; Bn, *Brassica napus* AFH41826.1; Ca, *Capsicum annuum* XP016574679.1; Cc, *Cajanus cajan* XP020219513.1; Cf, *Cardamine flexuosa* AGN29205.1; Cs, *Citrus sinensis* NP001275772.1; Dc, *D. carota* XP017232221.1 (DcSOC1a), XP017235334.1 (DcSOC1b), and XP017245184.1 (DcSOC1c); Eg, *Erythranthe guttata* XP012843635.1; Gh, *Gossypium hirsutum* AEA29618.1; Ha, *Helianthus annuus* XP022035849.1; Jr, *Juglans regia* XP018851690.1; Lc, *Litchi chinensis* AGS32267.1; Nt, *Nicotiana tabacum* NP001312958.1; Os, *Oryza sativa* Q9XJ60.1; Pm, *Prunus mume* XP008232833.1; Pp, *Prunus persica* XP007221064.2; Si, *Sesamum indicum* XP011091217.1; Tc, *Theobroma cacao* XP007051979.1; Vv, *Vitis vinifera* ACZ26527.1; Zm, *Zea mays* AIR75259.1.

We previously reported a preliminary study of the photoperiod pathway in carrot^42^. Only one *DcGI* sequence was retrieved from the carrot genome database and localized at chromosome (Chr.) 1, like *Dct293* (Supplementary Table S2). The nucleotide diversity (π) of *DcGI* in 21 DCS accessions and 13 DCC accessions was significantly lower than that in 5 Dau accessions (Fig. 1, Supplementary Table S1). *DcGI* endured selective pressures during evolution, according to the neutral tests of Tajima’s D (TD)^49^ and Fu and Li’s F (FF)^50^. Protein sequence alignment was performed to further explore their relationships with 27 *GI* homologues from other species. The tree was divided into four groups, and *DcGI* was clustered within group I and closely associated with *ChGIL*, *SiGIL*, and *EgGIL* (Fig. 2A).

Although 28 *CO*/*COL* sequences were retrieved, only 16 sequences had complete zinc finger B-box 1 and CCT domains^25^ (Supplementary Table S2). The size of the *DcCO* sequence was only 297 bp, without complete B-box 1 and CCT domains. *DcCOL2*, like *Dct43207*, had two tandem duplications and was localized at Chr. 2. *DcCOL4*, like *Dct43377*, was localized at Chr. 1. *DcCOL5* had two homologues and was localized at Chrs. 3 and 6, like *Dct7859* and *Dct20940*, respectively. *DcCOL13* also had two homologues and was localized at Chrs. 1 and 3, but only *DcCOL13b* was found in the transcriptome, like *Dct3283*. *DcCOL15*, like *Dct39974*, was localized at Chr. 7. Moreover, 1 *DcCOL9*, 3 *DcCOL*10, and 4 *DcCOL16* homologues were retrieved, which had not previously been reported in the transcriptome^42^. *DcCOL9* was localized at Chr. 3. Three *DcCOL10* homologues with 83.05% identity were localized at Chrs. 4, 5, and 6, respectively. Four *DcCOL16* homologues with 55.61% identity had only B-box 1 and CCT domains and were localized at Chrs. 3, 4, 7, and 9, respectively. Except for *DcCOL13b*, the nucleotide diversity (π) of the other *DcCOL*s in DCS and DCC was lower than that in Dau (Fig. 1). According to the neutrality tests of TD and FF, *DcCOL10a*/*c*, *DcCOL13a*, and *DcCOL16a*/*d* had significantly negative values, which indicated that these genes might be selected with a higher pressure during evolution. Additionally, it was interesting that *DcCOL5b*, *DcCOL11*, and *DcCOL*15 in DCS had significantly positive values, which indicated that these genes might be strongly selected during carrot evolution. Based on the phylogenetic analysis, 15 *DcCOL*s were divided into three groups (Fig. 2B). *DcCOL2*, *DcCOL4*, and *DcCOL5a/b* were clustered into group I, which were grouped with *AtCO*/*AtCOL1*/*2*, *AtCOL3*/*4*, and *AtCOL5*, respectively. *DcCOL16a*/*b*/*c*/*d* were clustered into group II and were closely associated with *AtCOL6* and *AtCOL16*. *DcCOL9*, *DcCOL10a*/*b*/*c*, *DcCOL13a*/*b*, and *DcCOL15* were assigned to group III and were closely associated with *AtCOL9*/*10*, *AtCOL13*, and *AtCOL14*/*15*, respectively.

**Figure 3:**
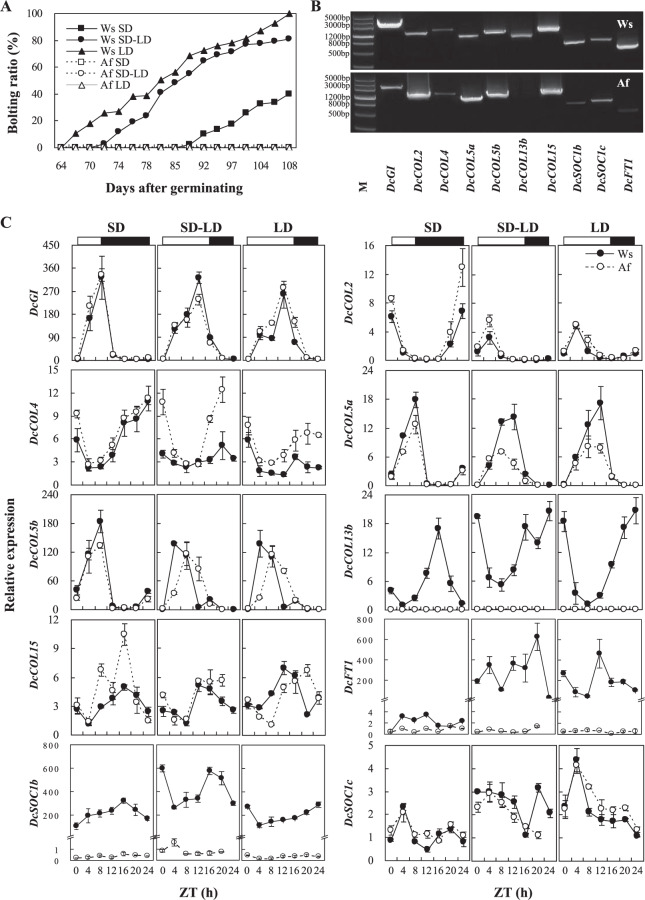
The circadian rhythm of *DcGI*, *DcCOL*s, *DcFT1*, and *DcSOC1*s under different photoperiods. **(A)** The bolting ratio of wild species Songzi (Ws) and orange cultivar Amsterdam forcing (Af) under different photoperiods. When the seedlings had 3–4 leaves and 37 days after germinating, the plants were subjected to 8 h light/16 h dark cycle treatment (as a short day (SD)), and to a 16 h light/8 h dark cycle, supplemented with white fluorescent light of 30 μmol/m^2^/s treatment (as a long day (LD)). After being treated for 27 days, half of the plants under SD conditions were subjected to LD conditions for 7 days (as SD-LD). There were about 12 days with a low temperature below 10 °C during the treatment. **(B**) Reverse transcript PCR of *DcGI*, *DcCOL2*, *DcCOL4*, *DcCOL5a*/*b*, *DcCOL13b*, *DcCOL15*, *DcSOC1b*/*c*, and *DcFT1* open reading frame (ORF) amplification products, respectively; M represents DNA Marker III. **(C**) The relative expression of *DcGI*, *DcCOL*s, *DcFT1*, and *DcSOC1*s under different photoperiods by real-time qPCR.

Four *DcFT* homologues were retrieved and annotated as *Hd3a*^48^, but only two sequences had the complete phosphatidylethanolamine-binding protein (PEBP) domain and were localized at Chrs. 1 and 7, respectively (Supplementary Table S2). Additionally, *DcFT1* had a 23.66 kbp large intron by compared its cDNA sequence with carrot genome^48^ (Fig. 3B), and *DcFT2* was the same as DcFT KY768910 (GenBank number)^47^. The nucleotide diversity (π) of *DcFT2* in DCS was significantly lower than that in DCC and Dau, and it might be selected during evolution, according to the neutrality tests of TD and FF (Fig. 1). Twenty-eight *FT*/*Hd3a* protein sequences of other species were aligned to explore these gene relationships. The phylogenetic tree was divided into four groups. *DcFT1* was assigned to group I and associated with *EuHd3a*, *LsFT*, and *DlFT1*, while *DcFT2* was assigned to group II and was closely associated with *VvFT*, *InFTL*, and *NtFTL* (Fig. 2C).

Six *DcSOC1* homologues were retrieved, but only four sequences had the complete MADS-box domain, K-box domain, and SOC1/MOTIF (Supplementary Table S2). *DcSOC1a* has not previously been reported and was localized at Chr. 2. *DcSOC1b*, like *Dct4069*, had two tandem duplications and was localized at Chr. 2. *DcSOC1c* was localized at Chr. 4, like *Dct34200*. For *DcSOC1a*, the nucleotide diversity (π) of DCS was significantly lower than that of DCC and Dau, while similar to that of *DcSOC1b*/*c* (Fig. 1). Twenty-two *SOC1* protein sequences of other species were aligned to explore the relationships. The phylogenetic tree was divided into four groups. *DcSOC1a*/*b*/*c* were assigned to group III and were associated with *HaSOC1L* (Fig. 2D).

### Circadian rhythm of *DcGI*, *DcCOL*s, *DcFT1*, and *DcSOC1*s under different photoperiods

Based on the sequences and phylogenetic analysis, *DcGI*, *DcCOL2*, *DcCOL4*, *DcCOL5a*/*b*, *DcCOL13b*, *DcCOL15*, *DcFT1*, and *DcSOC1b*/*c* were screened for a further study of the circadian rhythm. When the seedlings of Ws and Af had 3–4 leaves, the plants were subjected to SD and LD treatments for 27 days, respectively, and half of the seedlings under SD conditions were then subjected to the above LD conditions for 7 days (as SD-LD). Under LD conditions, Ws plants began to bolt for 67 days after germinating, and the bolting ratio reached 100% after 108 days. Under SD-LD and SD conditions, the bolting of Ws was delayed for approximately 5 and 22 days, and the bolting was 81% and 40% after 108 days, respectively. No bolting plant was found in Af under the above treatments (Fig. 3A). Except that of *DcCOL13b*, which was only expressed in Ws, the other gene open reading frame (ORF) regions were separately cloned from both Ws and Af (Fig. 3B).

The circadian patterns of *DcGI* expression in Ws and Af were similar to those described for *AtGI*^51^ and *OsGI*^15^ (Fig. 3C). Under SD conditions, the expression of *DcGI* began to increase from zeitgeber time (ZT) 0 to 8 and reached trough levels 4 h later, and its peak value was similar for Ws and Af (323.1/334.8). Under SD-LD conditions, the expression of *DcGI* peaked at ZT 12 and reached trough levels 8 h later, while the peak value (322.3) of *DcGI* in Ws was higher than that in Af (237.6). The *DcGI* expression pattern under LD conditions was similar to that under SD-LD conditions, and its peak value was similar for Ws and Af (256.7/281.8).

Under SD conditions, the expression of *DcCOL2* peaked at ZT 0 and then reached trough levels at ZT 8 in Ws and Af. Under SD-LD and LD conditions, the expression of *DcCOL2* peaked at ZT 4 and then declined to trough levels 4 h later. The peak values (6.8/3.2) of *DcCOL2* in Ws were lower than those in Af under both SD and SD-LD conditions (12.9/5.6), while they were similar under LD conditions (4.8/5.0). Under SD conditions, the expression patterns of *DcCOL4* were similar in Ws and Af (11.3/10.9) and peaked at ZT 0. Under SD-LD and LD conditions, the expression levels of *DcCOL4* in Ws (5.1/5.9) were significantly reduced, but were still higher in Af (12.4/7.7).

Under SD conditions, the circadian patterns of *DcCOL5a*/*b* were similar in Ws and Af and peaked at ZT 8. Under SD-LD and LD conditions, the peaks of *DcCOL5a* in Ws (14.2/17.1) shifted from ZT 8 to 12 and retained a similar level, but were reduced in Af (7.0/8.0). The peaks of *DcCOL5b* in Ws were advanced from ZT 8 to 4 under SD-LD and LD conditions, while those in Af were similar to those under SD conditions. *DcCOL13b* was only expressed in Ws, and not in Af. Under SD conditions, the expression of *DcCOL13b* peaked at ZT 16, but it peaked at ZT 0 under SD-LD and LD conditions. Under SD conditions, the expression level of *DcCOL15* in Af (10.5) was higher than that in Ws (5.0) and peaked at ZT 16. Under SD-LD and LD conditions, the peaks of *DcCOL15* in Ws (5.0/7.2) were shifted at ZT 12, while those in Af (5.7/6.7) reduced and shifted at ZT 20.

Under SD-LD and LD conditions, the expression levels of *DcFT1* in Ws (626.0/463.0) were about 179 and 132 times higher than that under SD conditions (3.5), while its expression in Af (1.4/0.7/1.4) constantly remained at low levels. The expression pattern of *DcSOC1b* was significantly different from that of *DcSOC1c*. The expression levels of *DcSOC1b* in Ws (322.3/290.0/599.0) were about 500, 610, and 371 times higher than those in Af under SD, SD-LD, and LD conditions (0.6/0.5/0.9), respectively. The expression patterns of *DcSOC1c* in Ws and Af were similar under the treatments.

### Trends of *DcGI*, *DcCOL*s, *DcFT1*, and *DcSOC1*s expressed in spring and autumn

**Figure 4:**
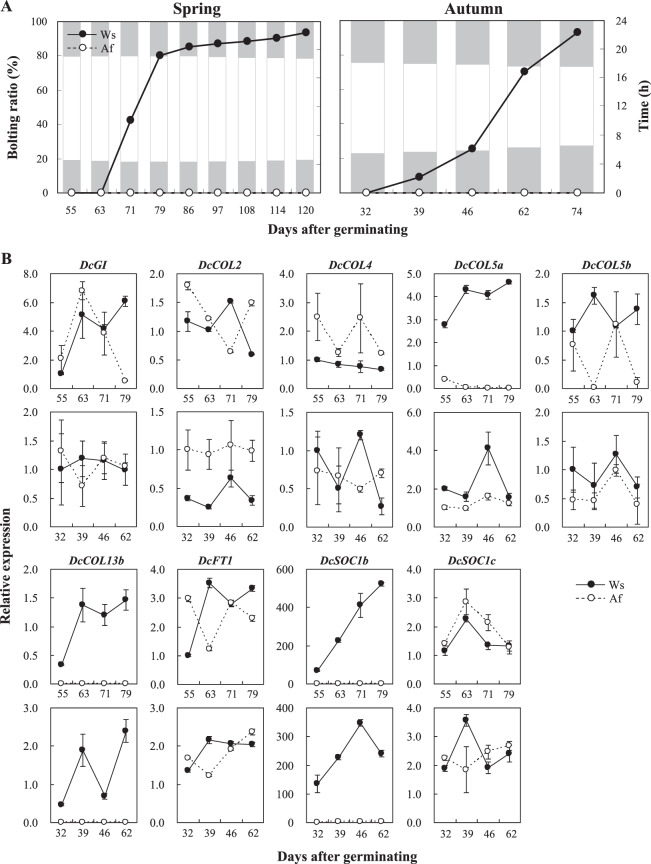
Relative expression levels of *DcGI*, *DcCOL*s, *DcFT1*, and *DcSOC1*s in spring and autumn. **(A)** The bolting ratio of Ws and Af. The seeds were sown in the field under natural photoperiods and temperature conditions on 19 March (spring) and 4 August (autumn), respectively. The day lengths were about 16 h in spring and about 14 h in autumn, when Ws began to bolt. The white and gray frame represented the sun rising and setting time in spring and autumn. There were about 15 days with a low temperature below 10 °C after the seeds had germinated in spring. **(B)** The relative expression levels of *DcGI*, *DcCOL*s, *DcFT1*, and *DcSOC1*s in Ws and Af in spring and autumn by real-time qPCR. The leaves from five plants were sampled from 08:00 to 09:00 about 55, 63, 71, and 79 days after germinating in spring, and about 32, 39, 46, and 62 days after germinating in autumn, when the plants had about five leaves.

Plants are usually induced to flower through two or three interconnected pathways under natural conditions^3,13,14^. In order to further understand the functions of *DcGI*, *DcCOL*s, *DcFT1*, and *DcSOC1*s during carrot growth, their expression levels were analyzed in Ws and Af in spring and autumn. The seeds were sown directly in the field under natural photoperiods and temperature conditions on 19 March (spring) and 4 August (autumn), respectively. In spring, Ws plants began to bolt for about 65 days after germinating and the bolting ratio rapidly reached 80.3% after 79 days after germinating, while in autumn, the bolting plants initiated 39 days after germinating and peaked at 93.3% after 74 days. No bolting plants were observed for Af during these two seasons (Fig. 4A).

The expression level of *DcGI* in Ws constantly increased, but later decreased in Af during spring, and fluctuated during autumn (Fig. 4B). *DcCOL2* in Af maintained a higher expression level than that in Ws at the beginning and fluctuated during spring, while it maintained a constantly higher level in Af than that in Ws during autumn. The expression of *DcCOL4* in Ws remained at low levels, but was high in Af during spring, while it fluctuated during autumn. The expression of *DcCOL5a* in Ws maintained a higher level than that in Af during both spring and autumn, but that of *DcCOL5b* was similar. *DcCOL13b* was only expressed in Ws and peaked at the middle stage. The expression patterns of *DcFT1* in Ws and Af were different and fluctuated over the course of the two seasons. *DcSOC1b* in Ws was expressed at a significantly higher level than that in Af during the two seasons, but *DcSOC1c* showed a similar expression level.

### Effect of the overexpression of *DcCOL2* and *DcCOL5a* on the flowering time of *Arabidopsis*

**Figure 5:**
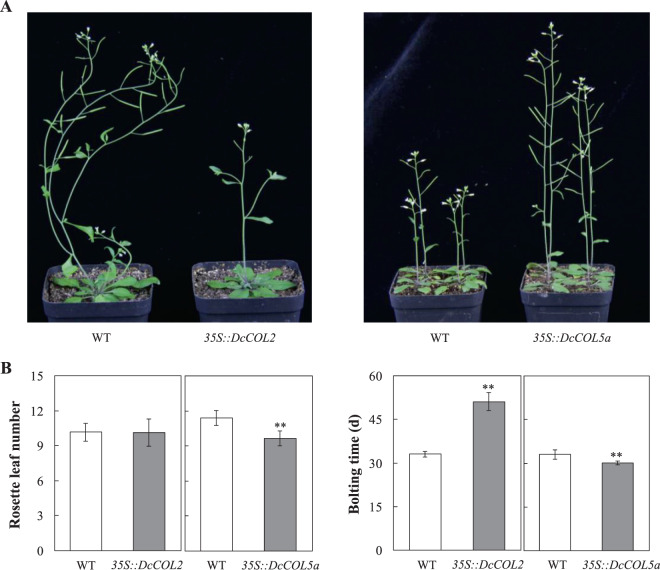
Bolting time of wild-type and transgenic *Arabidopsis* plants. **(A)** The morphology and bolting time of wild-type and transgenic *Arabidopsis* plants (T_2_), where *35 S::DcCOL2* and *35 S::DcCOL5a* represent transgenic plants. (**B**) The bolting time of wild-type and transgenic *Arabidopsis* plants (T_2_). Values are reported as the mean ± SE. Duncan’s post-hoc multiple comparison was used to detect significant differences. * and ** represent a 0.05 and 0.01 significance level.

To further understand the functions of *DcCOL2* and *DcCOL5a*, their sense cDNAs with the CaMV35S promoter were transformed into wild-type Columbia (WT) *Arabidopsis* plants (Supplementary Fig. S1A). In the 14 *35 S::DcCOL2* plants (T_1_), 13 were detected in the *DcCOL2* cDNA products, but one was not detected (Supplementary Fig. S1B). All 12 *35 S::DcCOL5a* plants (T_1_) were detected in the *DcCOL5* cDNA products. The bolting time of *35S::DcCOL2* plants (T_2_) was significantly delayed by about 18.1 days, but the rosette leaf number was similar to that of the WT (Fig. 5A,B). Conversely, the bolting time of *35 S::DcCOL5a* plants (T_2_) was slightly accelerated by 3.0 days and the rosette leaf number decreased by about 1.8.

## Discussion

For cultivated carrot, it takes almost a year for it to complete its lifecycle. Many endogenous and external factors can affect carrot bolting and flowering during growth^41–43^. Plants with 8–12 leaves are required to respond to a low temperature in carrot^41^, but some landraces and cultivars can initiate bolting after a short vernalization period^52,53^. During the winter–spring period or spring cultivation, the premature bolting of carrot might be interconnected by vernalization and photoperiod pathways^3,12–14^. A basic and putative flowering time network was suggested in carrot^42^, but knowledge of its photoperiodic regulation is limited. In order to further understand the molecular mechanisms of the photoperiod pathway, the establishment of a set of carrot materials with a sensitivity to different photoperiods is required. In this study, Ws plants began to bolt 67 days after germinating and reached 100% after 108 days under LD conditions (Fig. 3A). In spring, under natural conditions, Ws plants similarly began to bolt about 65 days after germinating, but the bolting ratio increased rapidly (Fig. 4A). Ws plants initiated bolting 39 days after germinating and rapidly reached 93.3% after 74 days in autumn (Fig. 4A), but there was no low temperature recorded during the growth. In addition, the bolting time of Ws could be delayed for 22 days and its bolting ratio was significantly reduced to 40% about 108 days after germinating (Fig. 3A), which was similar to a previous report showing that the floral initiation of carrot can be inhibited by SDs^42^. Meanwhile, the orange cultivar Af showed no bolting phenomenon, with tolerance of flowering characterization under different photoperiods (Figs. 3A and 4A). This result suggests that Ws and Af can be regarded as suitable materials for further studying photoperiodic flowering regulations.

According to the carrot genome^48^, previously reported de novo transcriptome genes^42^ could be localized to specific chromosomes and some new photoperiod genes were identified in this study, including 9 *DcCOL*s, 1 *DcFT*, and 1 *DcSOC1*, but there is no complete structure of *CO*^6,11,15–18^ (Supplementary Table S2). *GI* plays a general role in controlling circadian rhythms for flowering and is highly conserved in seed plants^16,19,54^. One *DcGI* was identified in *Daucus* species and was strongly selected during evolution (Fig. 1). The circadian pattern of *DcGI* is similar to that described for *AtGI*^19,54^ and *OsGI*^15^, and its peak could be delayed by a long photoperiod (Fig. 3C). However, the response for Af and Ws was different that the peak value was quickly reduced in Af under SD-LD conditions, but was maintained in Ws, which was consistent with the trend of expression in spring (Fig. 4B). According to the bolting ratio and time of Ws (Figs. 3A and 4A), *DcGI* might promote flowering in the carrot photoperiodic pathway.

*CO*/*COL* plays a central role in regulating the downstream florigen gene by integrating light and circadian clock signals^3,11,18,25,26^, and multiple homologues have been identified in many species^32–35^. Only some *CO* homologues in other species can be regarded as potential regulators of *FT*-like genes^12^. In this study, 15 *DcCOL*s were identified with complete B-box 1 and CCT domains in carrot (Supplementary Table S2, Fig. 2B). According to the nucleotide diversity (π) and neutrality tests, 8 *DcCOL*s might be selected during evolution, especially *DcCOL5b*, *DcCOL11*, and *DcCOL15* in cultivars (Fig. 1). *DcCOL2* showed a similar morning-phase rhythm in Ws and Af, which was comparable to *AtCOL2*^28^ and *ClCOL2*^55^. In Ws, the peak of *DcCOL2* was advanced and higher under SD conditions than under SD-LD and LD conditions (Fig. 3C), but the bolting time was significantly delayed and the ratio was reduced under SD conditions (Fig. 3A). In Af, the patterns of *DcCOL2* were similar and the peak value was higher than that in Ws (Fig. 3C). In the two seasons, the expression of *DcCOL2* in Af showed higher levels than in Ws, but no bolting plants were found in Af (Fig. 4A). The bolting time of *35 S::DcCOL2 Arabidopsis* plants (T_2_) was also significantly delayed, but its rosette leaf number was similar to that of the WT (Fig. 5). *DcCOL4* also had a morning expression pattern and peaked at dawn under SDs, similar to *DcCOL2*. It was strongly repressed under a long photoperiod in Ws, but it could maintain a constantly high level in Af (Fig. 3C), even during spring (Fig. 4B). A recent study showed that an increase of *COL4* expression causes flowering delay in *Arabidopsis*^29^, but *COL2* has little effect on the flowering time^28^. Maintaining a higher expression of *DcCOL2* and *DcCOL4* might be related to the bolting tolerance in Af, but this claim needs more supporting evidence.

Two *DcCOL5* homologues were identified and their circadian rhythms under SDs were similar to that of *ClCOL5*^55^, but their responses to a long photoperiod were different (Fig. 3C). It was obvious that the peak of *DcCOL5a* was postponed and maintained a similar level under a long photoperiod, but that of *DcCOL5b* was advanced and reduced in Ws. In Af, the expression of *DcCOL5a* was downregulated, but there was no *DcCOL5b* response under SD-LD and LD conditions. In the two seasons, the expression level of *DcCOL5a* in Ws was constantly higher than that in Af, but the expression levels of *DcCOL5b* fluctuated (Fig. 4B). The bolting time of *35 S::DcCOL5a Arabidopsis* plants (T_2_) was only slightly accelerated (Fig. 5). *DcCOL13b* was only expressed in Ws and maintained similar expression levels under different photoperiods (Fig. 3C), but its circadian rhythm presented contrasting patterns under SD and SD-LD/LD conditions, similar to that of *OsCOL13*^36^. Whether *DcCOL13b* represses flowering or not requires further investigation.

*FT* is the major primary target of *CO* in leaves^4,6,7,12^. In the carrot genome, there are two *FT* homologues localized at Chrs. 1 and 7, respectively^48^ (Supplementary Table S2). According to the phylogenetic analysis, *DcFT1* was assigned to group I and associated with *EuHd3a*, *LsFT*, and *DlFT1*, while *DcFT2* was assigned to group II and closely associated with *VvFT*, *InFTL*, and *NtFTL* (Fig. 2C). It is interesting that *DcFT1* had a 23.66 kbp large intron by compared its cDNA sequence with the carrot genome^48^ (Fig. 3B). The circadian rhythm of *DcFT1* in Ws was similar to that of *AtFT* and its expression levels were significantly upregulated under SD-LD/LD conditions, but constantly low in Af^4,7^ (Fig. 3C). The expression patterns of *DcFT1* in Ws and Af were different, but the levels were similar during the two seasons (Fig. 4B). The expanded studies show that *FT* homologues have a conserved function in promoting flowering^12^. Different responses of *DcFT1* in Ws and Af might be related to the different bolting response, which could give a clue for further studying its function in flowering regulation.

*SOC1* integrates multiple flowering signals to interact with multiple MADS-box proteins and regulate the expresssion of flowering genes^8,9,21,56,57^. Three *DcSOC1s* with complete structures were identified and found to be highly conserved during evolution (Fig. 1). The circadian rhythm patterns of *DcSOC1b*/*c* were different under the three photoperiod treatments (Fig. 3C). The expression of *DcSOC1b* in Ws maintained significantly higher levels than those in Af, while *DcSOC1c* showed similar levels. The same results were observed in the two studied seasons (Fig. 4B). These results suggest that *DcSOC1b* might play a role in regulating downstream flowering genes, like *AtSOC1*^9,20^ and *ZmSOC1*^57^.

In general, Ws plants could initiate bolting 39 days after germinating without low-temperature treatment, which suggests that it can be regarded as a suitable material for studying photoperiodic flowering regulation. Based on previous studies, 1 *DcGI*, 15 *DcCOL*s, 2 *DcFT*s, and 3 *DcSOC1*s were identified in the photoperiod pathway. The circadian rhythm peaks of *DcGI*, *DcCOL2*, *DcCOL5a*, and *DcCOL13b* could be delayed under LD conditions. The peak value of *DcCOL2* in Af was significantly higher than that in Ws under SD conditions, and their values could be reduced under SD-LD/LD conditions. Under the photoperiod treatments, the peak values of *DcCOL5a* in Ws were constantly higher than those in Af, even during the two seasons. The expression levels of *DcFT1* in Ws were significantly upregulated under SD-LD/LD conditions compared with those in Af. These responses of *DcCOL2*, *DcCOL5a*, and *DcFT1* might be related to the different bolting responses of Ws and Af. This study could provide valuable information that increases our understanding of the key integrator genes in the carrot photoperiod pathway. Future work focusing on characterizing the function of these candidate genes will be helpful for screening those accessions with tolerance to premature bolting.

## Methods

### Retrieval and phylogenetic analysis of *DcGI*, *DcCOL*, *DcFT*, and *DcSOC1* sequences in carrot

*GI*, *COL*, *FT*, and *SOC1* are the key transcript regulators in the photoperiodic pathway^4,6,7,11,16,17^. Based on the carrot genome^48^, the sequences of 1 *DcGI*, 28 *DcCOL*, 4 *DcFT*, and 6 *DcSOC1* homologues were retrieved and aligned with transcriptome unigenes^42^ using DNAMAN software (Supplementary Table S1). Twenty-seven *GI*, 22 *CO/COL*, 28 *FT/Hd3a*, and 22 *SOC1* homologue protein sequences of other species were retrieved from the NCBI database and used to perform a phylogenetic analysis with the abovementioned genes through MEGA5.0 using the neighbor-joining method, respectively. The phylogenetic tree architecture was validated using the probabilistic bootstrap test with 1000 replicates.

### Nucleotide diversity and selection of *DcGI*, *DcCOL*s, *DcFT*s, and *DcSOC1*s in *Daucus* species

Twenty-one *D. carota* var. *sativus* accessions (as DCS), 4 *D. carota* subsp. *gummifer* species and 9 *D. carota* subsp. *carota* species (as DCC), and 5 *Daucus* species (as Dau) were used to perform the evolutionary analysis of *GI*, *COL*, *FT*, and *SOC1* homologues (Supplementary Table S1). The genome of cultivar Af, breeding lines 17166 C, 170P2A, 17P25A, and wild species Ws were re-sequenced with each leaf sample in the Illumina HiSeq. 2500 sequencing platform (Biomarker Technologies Co., Ltd. Beijing, China). The sequence data of 34 accessions were downloaded from NCBI (https://www.ncbi.nlm.nih.gov/genome/?term=carrot)^48^. All genome sequence reads from 39 individual accessions were trimmed and filtered prior to analysis, and aligned to the carrot genome^48^ using BWA software^58^. Alignment files were converted into SAM/BAM files and simple nucleotide polymorphism (SNP) calling was performed using SAMtools^58^. Low-quality SNPs with a base quality value of <20 and a read depth of <4× or those with >32× coverage from the sequences were excluded because these SNPs may be false positives. The FASTA sequences of 39 individuals were extracted using BCFtools^59^. Nucleotide diversity (π) for the genomic sequence of each data group and neutral tests of TD^49^ and FF^50^ were estimated based on the neutral model prediction by DnaSP 6^60^.

### Gene cloning and circadian rhythm analysis of *DcGI*, *DcCOL*s, *DcFT1*, and *DcSOC1*s

For the circadian rhythm analysis, Ws and Af seeds were sown in flats (90 cm × 30 cm × 30 cm) filled with a 1:2:3 mixture of soil, vermiculite, and turf in the greenhouse at a temperature of 13–16/25–28 °C night/day in late February. The average maximum light intensity per day in the greenhouse (55.4 ± 3.7 W ∙ m^−2^) was only 30.6% of that in the field (180.9 ± 14.4 W ∙ m^−2^). When the seedlings had 3–4 leaves and 37 days after germinating, the plants were moved to the field under a natural temperature and subjected to SD (8 h light/16 h dark cycle) and LD (16 h light/8 h dark cycle, supplemented with white fluorescent light of 30 μmol/m^2^/s) treatments for 27 days. Then, half of the seedlings that had 6–7 leaves under SD treatment were subjected to the above LD conditions for 7 days (as SD-LD), and half remained under SD conditions. During the treatment, there were about 12 days with a low temperature (below 10 °C)^41^. The leaves of each treatment were sampled from five plants with three biological replicates at 4 h intervals after dawn at zeitgeber time 0 (ZT 0). All samples were immediately frozen in liquid nitrogen and stored at −80 °C until use. After this, all plants were grown under natural conditions, and the number of bolting plants was counted every 2–4 days until 108 days after germinating.

The candidate genes of *DcGI*, *DcCOL2*, *DcCOL4*, *DcCOL5a*/*b*, *DcCOL13b*, *DcCOL15*, *DcFT1*, and *DcSOC1b*/*c* were selected to study the circadian rhythm under different photoperiod treatments. The specific primers for the open reading frame (ORF) sequence clone and real-time qPCR were designed using Primer Premier 5.0 based on the abovementioned gene sequences (Supplementary Table S3). The operation procedures for RNA extraction, cDNA synthesis, reverse transcription PCR, and real-time qPCR were carried out as described by Wang *et al*.^61^. *Tublin* was amplified along with the target genes as an endogenous control to normalize the expression levels between samples^62^. Three biological repetitions were performed for each expression data point. The comparative C_T_ (2^−ΔΔCt^) method was applied for calculation^63^. The lowest expression level of each gene was used for calibration. Values are reported as the mean ± SE from the replicates.

### Expression analysis of *DcGI*, *DcCOL*s, *DcFT1*, and *DcSOC1*s in spring and autumn

The expression levels of *DcGI*, *DcCOL*s, *DcFT1*, and *DcSOC1*s were further analyzed to understand their functions during carrot growth under natural conditions. The seeds of Ws and Af were sown with 20 cm row spacing in the field under natural photoperiods and temperature conditions on 19 March (spring) and 4 August (autumn), respectively. The cultivation and management of carrot referred to that of Rubatzky *et al*.^45^. There were about 15 days with a low temperature (below 10 °C) after the seeds had germinated in spring and no low temperature was recorded before the plants had bolted in autumn. When the plants had about five leaves, the leaves from five plants were sampled with three biological replicates from 08:00 to 09:00 at 55, 63, 71, and 79 days after germinating in spring, and at 32, 39, 46, and 62 days after germinating in autumn. The number of bolting plants was also investigated. Sample collection, total RNA extraction, cDNA synthesis, gene expression, and data analysis were performed as described by Wang *et al*.^61^.

### Flowering time of transgenic *Arabidopsis* with *DcCOL2* and *DcCOL5a*

To further understand the functions of *DcCOL2* and *DcCOL5a*, their full-length cDNAs were amplified using gene-specific primers and subcloned into the vector PBI121 plasmid and agrobacterium GV3101 (Tiangen, China) to generate *35 S::DcCOL2* and *35 S::DcCOL5a*, which were introduced into the wild-type Columbia (WT) *Arabidopsis* by the floral dip method^64^. Transgenic plants were screened on Murashige and Skoog (MS) agar plates supplemented with 50 mg/L kanamycin. After being left in darkness at 4 °C for 3 days, kanamycin-resistant seedlings were transferred to soil after 10 days and grown in a controlled growth room (~22 °C, 16 h light/8 h dark). The seeds from T_0_ plants were harvested and sown on MS plates supplemented with 50 mg/L kanamycin and treated under the above-described conditions. The seedlings (T_1_) were transferred and grown under the above-described conditions. Fifteen *35 S::DcCOL2* and 13 *35 S::DcCOL5a* plant leaves were independently sampled and tested using PCR. The PCR program was followed as described by Wang *et al*.^61^. WT and T_2_ transgenic plants were grown under the abovementioned conditions to investigate the bolting time and the number of rosette leaves^30^. Values are shown as the mean ± SE from the replicates. Duncan’s post-hoc multiple comparison was used to detect significant differences in the number of rosette leaves and bolting time between wild-type and transgenic *Arabidopsis* plants (T_2_) using SPSS (version 10.0).

## Supplementary information

Supplementary information.
