# Supplementary material for: The Response of COL and FT Homologues to Photoperiodic Regulation in Carrot (Daucus carota L.)
Source: Sci Rep. 2020 Jun 19;10:9984. doi: 10.1038/s41598-020-66807-y (PMC7305175; doi:10.1038/s41598-020-66807-y)
Supplement: Supplementary file 1 — Supplementary information. [file 41598_2020_66807_MOESM1_ESM.doc]

**The Response of *COL* and *FT* Homologues to Photoperiodic Regulation in Carrot (*Daucus carota* L.)**

**Running title: Response of *COL* and *FT* Homologues in Carrot**

Lijie Liu^1+^, Chenggang Ou^1+^, Shumin Chen^1^, Qi Shen^1^, Bo Liu^1^, Min Li^1^, Zhiwei Zhao^1^, Xiaoping Kong^2^, Xiangping Yan^3^, & Feiyun Zhuang^1,3^*

^1^*Key Laboratory of Horticultural Crop Biology and Germplasm Innovation, Ministry of Agriculture; Institute of Vegetables and Flowers, Chinese Academy of Agricultural Science*

*No. 12 Nanda Street, Zhongguan Cun, Haidian District, Beijing 100081, China*

Lijie Liu, keji678123@126.com

Chenggang Ou, ouchenggang@caas.cn

Shumin Chen, chenshumin@caas.cn

Qi Shen, 1219198095@qq.com

Bo Liu, [731138090@qq.com](mailto:731138090@qq.com)

Min Li, li1504850091@163.com

Zhiwei Zhao, zhaozhiwei@caas.cn

Feiyun Zhuang*, corresponding author, Tel: 086-10-82105950, Fax: 086-10-62174123, [zhuangfeiyun@caas.cn](mailto:zhuangfeiyun@caas.cn)

^2^*College of Ecological Environment and Resources, Qinghai Nationalities University*

*No. 3, Bayi Middle Road, Chengdong District, Xining, Qinghai Province，810007*

Xiaoping Kong, [xnsc_kong@126.com](mailto:xnsc_kong@126.com)

^3^*Xining Institute of Vegetables, Xining*

*No. 4 Weisan Road, Biological Industry Park, Xining 810016, Qinghai*

Xiangping Yan, xnsc_xiang@126.com

**Supplementary Figure**


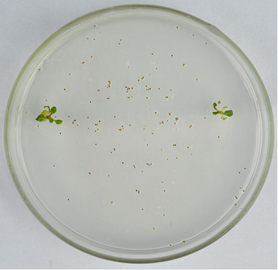


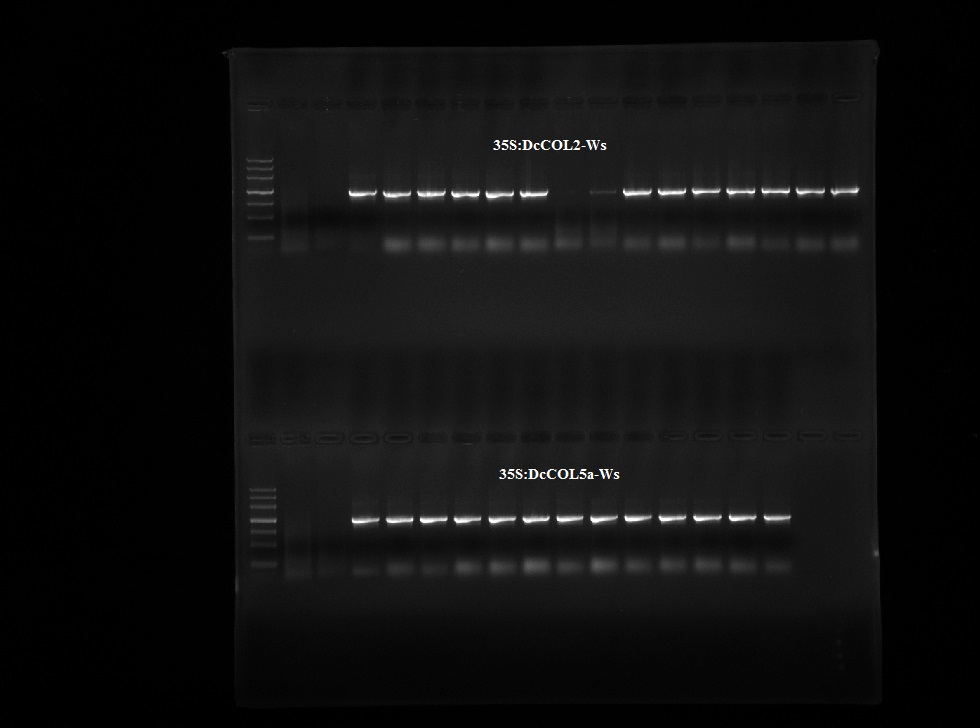

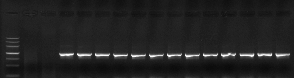


**A**

**B**

*35S::DcCOL2* (T_1_)

1

2

3

4

5

6

7

8

9

10

M

a

b

*35S::DcCOL5a* (T_1_)

11

12

13

14

WT

1200bp

1200bp

**Supplementary Fig. 1 Selection and identification of the transgenic*Arabidopsis* plants**

**A** Selection in MS media with kanamycin of the transgenic *Arabidopsis* seedlings (T_1_). **B** PCR identification of the transgenic *Arabidopsis* seedlings (T_1_), M represents DNA marker III; WT, a and b represent wild-type Columbia, ddH_2_O and transgene vector, respectively; 1 to 14 are transgenic plants.

**Supplementary materials**


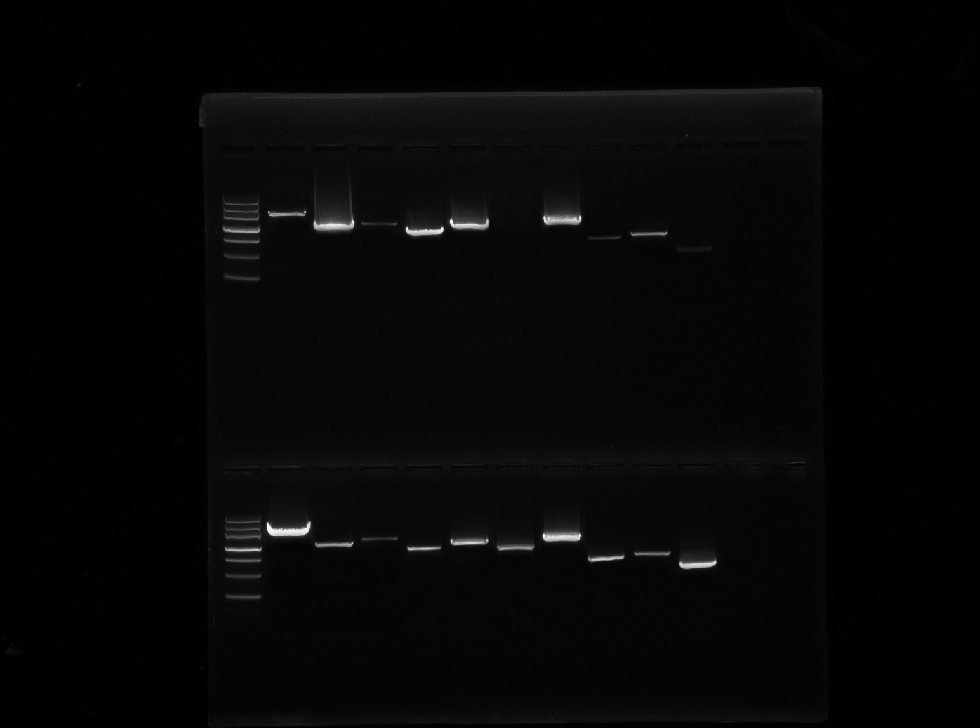


**Af**

**Ws**

**M**

***DcGI***

***DcCOL2***

***DcCOL4***

***DcCOL5a***

***DcCOL5b***

***DcCOL13b***

***DcCOL15***

***DcSOC1b***

***DcSOC1c***

***DcFT1***

**Fig. 3B** **Full-length gels of Reverse transcrispt PCR.**

M represents DNA Marker III.


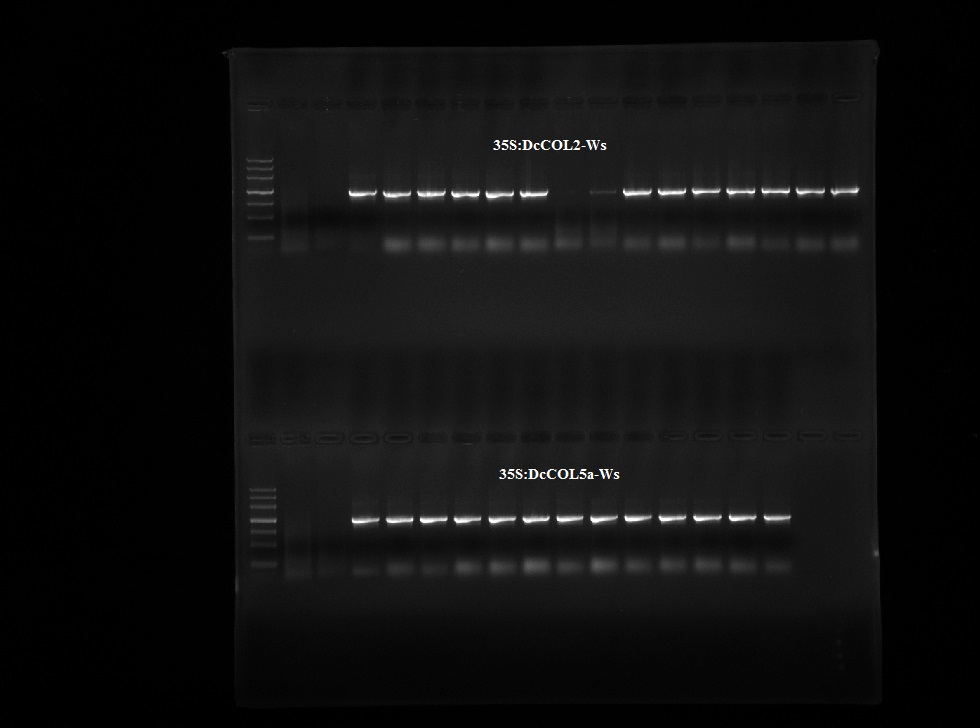


**Supplementary Fig. 1B Full-length gels**

**Supplementary Table S1** The list of 39 accessions

| Codes | Accessions | Sequences sources | Populations |
| --- | --- | --- | --- |
| 1 | *Daucus carota* var. *sativus* cultivar Amsterdam forcing (Af) | IVF-CAAS | DCS |
| 2 | *Daucus carota* var. *sativus* breeding line 17166C | IVF-CAAS | DCS |
| 3 | *Daucus carota* var. *sativus* breeding line 170P2A | IVF-CAAS | DCS |
| 4 | *Daucus carota* var. *sativus* breeding line 17P25A | IVF-CAAS | DCS |
| 5 | *Daucus carota* var. *sativus* breeding line B2566B | NCBI | DCS |
| 6 | *Daucus carota* var. *sativus* breeding line B6274B | NCBI | DCS |
| 7 | *Daucus carota* var. *sativus* breeding line B7262B | NCBI | DCS |
| 8 | *Daucus carota* var. *sativus* cultivar Brasilia | NCBI | DCS |
| 9 | *Daucus carota* var. *sativus* cultivar Chantenay Red Cored, accession PI 264232 | NCBI | DCS |
| 10 | *Daucus carota* var. *sativus* cultivar Kokubu, accession PI 261648 | NCBI | DCS |
| 11 | *Daucus carota* var. *sativus* cultivar Lange gele stomper, accession PI 451755 | NCBI | DCS |
| 12 | *Daucus carota* var. *sativus* cultivar Long Imperator 58, accession PI 632391 | NCBI | DCS |
| 13 | *Daucus carota* var. *sativus* cultivar Nantes No. 1, accession PI 187235 | NCBI | DCS |
| 14 | *Daucus carota* var. *sativus* cultivar Ping Ding, accession PI 652188 | NCBI | DCS |
| 15 | *Daucus carota* var. *sativus* cultivar Shin Kuroda Gosun, accession PI 652136 | NCBI | DCS |
| 16 | *Daucus carota* var. *sativus* cultivar White Belgian, accession PI 643114 | NCBI | DCS |
| 17 | *Daucus carota* var. *sativus* land race, accession PI 200876 | NCBI | DCS |
| 18 | *Daucus carota* var. *sativus* land race, accession PI 211590 | NCBI | DCS |
| 19 | *Daucus carota* var. *sativus* land race, accession PI 540422 | NCBI | DCS |
| 20 | *Daucus carota* var. *sativus* land race, accession PI 652336 | NCBI | DCS |
| 21 | *Daucus carota* var. *sativus* land race, accession PI 652374 | NCBI | DCS |
| 22 | *Daucus carota* subsp. *gummifer* accession Ames 26381 | NCBI | DCC |
| 23 | *Daucus carota* subsp. *gummifer* accession Ames 26383 | NCBI | DCC |
| 24 | *Daucus carota* subsp. *gummifer* accession Ames 31194 | NCBI | DCC |
| 25 | *Daucus carota* subsp. *gummifer* accession PI 478883 | NCBI | DCC |
| 26 | *Daucus carota* subsp. *carota* accession Ws | IVF-CAAS | DCC |
| 27 | *Daucus carota* subsp. *carota* accession Ames 26408 | NCBI | DCC |
| 28 | *Daucus carota* subsp. *carota* accession Ames 27395 | NCBI | DCC |
| 29 | *Daucus carota* subsp. *carota* accession PI 274297 | NCBI | DCC |
| 30 | *Daucus carota* subsp. *carota* accession PI 478369 | NCBI | DCC |
| 31 | *Daucus carota* subsp. *carota* accession PI 478861 | NCBI | DCC |
| 32 | *Daucus carota* subsp. *carota* accession PI 502244 | NCBI | DCC |
| 33 | *Daucus carota* subsp. *carota* accession PI 652358 | NCBI | DCC |
| 34 | *Daucus carota* subsp. *carota* accession PI 652393/SRX1134198 | NCBI | DCC |
| 35 | *Daucus guttatus* accession PI 652233 | NCBI | Dau |
| 36 | *Daucus sahariensis* accession Ames 29096 | NCBI | Dau |
| 37 | *Daucus syrticus* accession Ames 29108 | NCBI | Dau |
| 38 | *Daucus aureus* accession PI 319403 | NCBI | Dau |
| 39 | *Daucus capillifolius* accession PI 279764 | NCBI | Dau |

Note: IVF-CAAS, the institute of vegetables and flowers, Chinese Academy of Agricultural Sciences. These five accessions were re-sequenced with each leaf samples in Illumina HiSeq 2500 sequencing platform (Biomarker Technologies Co., Ltd. Beijing, China). NCBI, the genome sequences reads of 34 accessions were downloaded from https://www.ncbi.nlm.nih. gov/genome/?term=carrot (Iorizzo et al. 2016).

**Supplementary Table S2**

**Homologous of *GI*, *CO/COL*, *FT* and *SOC1* retrieved from the carrot genome ^a^**

| [**Gene**](https://www.ncbi.nlm.nih.gov/genome/proteins/860?genome_assembly_id=276486) | [**Gene ID**](https://www.ncbi.nlm.nih.gov/genome/proteins/860?genome_assembly_id=276486) | [**Chr.**](https://www.ncbi.nlm.nih.gov/genome/proteins/860?genome_assembly_id=276486) | [**Start**](https://www.ncbi.nlm.nih.gov/genome/proteins/860?genome_assembly_id=276486) | [**Stop**](https://www.ncbi.nlm.nih.gov/genome/proteins/860?genome_assembly_id=276486) | [**Protein product**](https://www.ncbi.nlm.nih.gov/genome/proteins/860?genome_assembly_id=276486) | [**Length**](https://www.ncbi.nlm.nih.gov/genome/proteins/860?genome_assembly_id=276486) | **Unigene** |
| --- | --- | --- | --- | --- | --- | --- | --- |
| *GI* | [108202572](https://www.ncbi.nlm.nih.gov/gene/108202572) | 1 | 12735909 | 12743314 | [XP017226526.1](https://www.ncbi.nlm.nih.gov/protein/XP_017226526.1) | 1166 | *Dct293* |
| ^▲^*CO* | [108198645](https://www.ncbi.nlm.nih.gov/gene/108198645) | 1 | 969982 | 971456 | [XP017221909.1](https://www.ncbi.nlm.nih.gov/protein/XP_017221909.1) | 297 |  |
| ^▲^*COL1* | [108214788](https://www.ncbi.nlm.nih.gov/gene/108214788) | 3 | 2615075 | 2618670 | [XP017242473.1](https://www.ncbi.nlm.nih.gov/protein/XP_017242473.1) | 266 |  |
| *COL2* | [108205797](https://www.ncbi.nlm.nih.gov/gene/108205797) | 2 | 35094334 | 35096417 | [XP017231361.1](https://www.ncbi.nlm.nih.gov/protein/XP_017231361.1) | 393 | *Dct43207* |
| *COL2* | [108205835](https://www.ncbi.nlm.nih.gov/gene/108205835) | 2 | 35103027 | 35105110 | [XP017231417.1](https://www.ncbi.nlm.nih.gov/protein/XP_017231417.1) | 393 | *Dct43207* |
| ^▲^*COL2* | [108193559](https://www.ncbi.nlm.nih.gov/gene/108193559) | 7 | 35020142 | 35022133 | [XP017215757.1](https://www.ncbi.nlm.nih.gov/protein/XP_017215757.1) | 346 |  |
| ^▲^*COL2* | [108195069](https://www.ncbi.nlm.nih.gov/gene/108195069) | 7 | 34994151 | 34995590 | [XP017217491.1](https://www.ncbi.nlm.nih.gov/protein/XP_017217491.1) | 170 |  |
| *COL4* | [108205134](https://www.ncbi.nlm.nih.gov/gene/108205134) | 1 | 35164821 | 35166472 | [XP017230434.1](https://www.ncbi.nlm.nih.gov/protein/XP_017230434.1) | 389 | *Dct43377* |
| ^▲^*COL4* | [108206789](https://www.ncbi.nlm.nih.gov/gene/108206789) | 2 | 39488835 | 39489323 | [XP017232684.1](https://www.ncbi.nlm.nih.gov/protein/XP_017232684.1) | 162 |  |
| *COL5a* | [108210932](https://www.ncbi.nlm.nih.gov/gene/108210932) | 3 | 12329844 | 12331169 | [XP017237885.1](https://www.ncbi.nlm.nih.gov/protein/XP_017237885.1) | 367 | *Dct7859* |
| *COL5b* | [108224798](https://www.ncbi.nlm.nih.gov/gene/108224798) | 6 | 34089534 | 34090672 | [XP017255010.1](https://www.ncbi.nlm.nih.gov/protein/XP_017255010.1) | 346 | *Dct20940* |
| ^▲^*COL5* | [108208509](https://www.ncbi.nlm.nih.gov/gene/108208509) | 2 | 20894586 | 20895085 | [XP017234532.1](https://www.ncbi.nlm.nih.gov/protein/XP_017234532.1) | 123 |  |
| ^▲^*COL6* | [108210393](https://www.ncbi.nlm.nih.gov/gene/108210393) | 3 | 34210954 | 34213064 | [XP017237142.1](https://www.ncbi.nlm.nih.gov/protein/XP_017237142.1) | 294 |  |
| ^▲^*COL9* | [108214870](https://www.ncbi.nlm.nih.gov/gene/108214870) | 1 | 45276299 | 45276766 | [XP017242597.1](https://www.ncbi.nlm.nih.gov/protein/XP_017242597.1) | 155 |  |
| *COL9* | [108211305](https://www.ncbi.nlm.nih.gov/gene/108211305) | 3 | 936003 | 939034 | [XP017238354.1](https://www.ncbi.nlm.nih.gov/protein/XP_017238354.1) | 401 |  |
| *COL10a* | [108215719](https://www.ncbi.nlm.nih.gov/gene/108215719) | 4 | 35688312 | 35691110 | [XP017243761.1](https://www.ncbi.nlm.nih.gov/protein/XP_017243761.1) | 404 |  |
| *COL10b* | [108223951](https://www.ncbi.nlm.nih.gov/gene/108223951) | 5 | 40868178 | 40871151 | [XP017253919.1](https://www.ncbi.nlm.nih.gov/protein/XP_017253919.1) | 409 |  |
| *COL10c* | [108193056](https://www.ncbi.nlm.nih.gov/gene/108193056) | 6 | 26002966 | 26006388 | [XP017215113.1](https://www.ncbi.nlm.nih.gov/protein/XP_017215113.1) | 405 |  |
| ^▲^*COL11* | [108223436](https://www.ncbi.nlm.nih.gov/gene/108223436) | 5 | 41686847 | 41688751 | [XP017253195.1](https://www.ncbi.nlm.nih.gov/protein/XP_017253195.1) | 426 |  |
| ^▲^*COL11* | [108226211](https://www.ncbi.nlm.nih.gov/gene/108226211) | 6 | 25241875 | 25243506 | [XP017256646.1](https://www.ncbi.nlm.nih.gov/protein/XP_017256646.1) | 357 |  |
| ^▲^*COL12* | [108211874](https://www.ncbi.nlm.nih.gov/gene/108211874) | 3 | 10220698 | 10221939 | [XP017239071.1](https://www.ncbi.nlm.nih.gov/protein/XP_017239071.1) | 249 |  |
| *COL13a* | [108204696](https://www.ncbi.nlm.nih.gov/gene/108204696) | 1 | 32923859 | 32926610 | [XP017229749.1](https://www.ncbi.nlm.nih.gov/protein/XP_017229749.1) | 376 |  |
| *COL13b* | [108215337](https://www.ncbi.nlm.nih.gov/gene/108215337) | 3 | 46723303 | 46725868 | [XP017243283.1](https://www.ncbi.nlm.nih.gov/protein/XP_017243283.1) | 390 | *Dct3283* |
| ^▲^*COL13* | [108220459](https://www.ncbi.nlm.nih.gov/gene/108220459) | 5 | 28806952 | 28810289 | [XP017249726.1](https://www.ncbi.nlm.nih.gov/protein/XP_017249726.1) | 379 |  |
| *COL15* | [108196522](https://www.ncbi.nlm.nih.gov/gene/108196522) | 7 | 17531506 | 17534445 | [XP017219331.1](https://www.ncbi.nlm.nih.gov/protein/XP_017219331.1) | 464 | *Dct39974* |
| *COL16a* | [108210639](https://www.ncbi.nlm.nih.gov/gene/108210639) | 3 | 37286865 | 37288139 | [XP017237500.1](https://www.ncbi.nlm.nih.gov/protein/XP_017237500.1) | 424 |  |
| *COL16b* | [108216126](https://www.ncbi.nlm.nih.gov/gene/108216126) | 4 | 15277638 | 15279231 | [XP017244294.1](https://www.ncbi.nlm.nih.gov/protein/XP_017244294.1) | 442 |  |
| *COL16c* | [108196488](https://www.ncbi.nlm.nih.gov/gene/108196488) | 7 | 3762394 | 3763862 | [XP017219277.1](https://www.ncbi.nlm.nih.gov/protein/XP_017219277.1) | 417 |  |
| *COL16d* | [108200187](https://www.ncbi.nlm.nih.gov/gene/108200187) | 9 | 21574981 | 21576788 | [XP017223731.1](https://www.ncbi.nlm.nih.gov/protein/XP_017223731.1) | 397 |  |
| ^▲^*FT* | [108195181](https://www.ncbi.nlm.nih.gov/gene/108195181) | 1 | 20669041 | 20666536 | [XP017217625.1](https://www.ncbi.nlm.nih.gov/protein/XP_017217625.1) | 153 |  |
| ^▲^*FT* | [108201625](https://www.ncbi.nlm.nih.gov/gene/108201625) | 1 | 20742289 | 20740967 | [XP017225405.1](https://www.ncbi.nlm.nih.gov/protein/XP_017225405.1) | 110 |  |
| *FT1* | [108201615](https://www.ncbi.nlm.nih.gov/gene/108201615) | 1 | 20716274 | 20692614 | [XP017225396.1](https://www.ncbi.nlm.nih.gov/protein/XP_017225396.1) | 177 |  |
| *FT2* | [108194504](https://www.ncbi.nlm.nih.gov/gene/108194504) | 7 | 6697534 | 6694985 | [XP017216959.1](https://www.ncbi.nlm.nih.gov/protein/XP_017216959.1) | 175 | KY768910 |
| *SOC1a* | [108206428](https://www.ncbi.nlm.nih.gov/gene/108206428) | 2 | 29660190 | 29646179 | [XP017232221.1](https://www.ncbi.nlm.nih.gov/protein/XP_017232221.1) | 218 |  |
| *SOC1b* | [108209100](https://www.ncbi.nlm.nih.gov/gene/108209100) | 2 | 43225705 | 43217222 | [XP017235334.1](https://www.ncbi.nlm.nih.gov/protein/XP_017235334.1) | 217 | *Dct40692* |
| *SOC1b* | [108209496](https://www.ncbi.nlm.nih.gov/gene/108209496) | 2 | 43279439 | 43272991 | [XP017235918.1](https://www.ncbi.nlm.nih.gov/protein/XP_017235918.1) | 217 | *Dct40692* |
| *SOC1c* | [108216842](https://www.ncbi.nlm.nih.gov/gene/108216842) | 4 | 5067881 | 5060723 | [XP017245183.1](https://www.ncbi.nlm.nih.gov/protein/XP_017245183.1) | 211 | *Dct34200* |
| ^▲^*SOC1* | [108217739](https://www.ncbi.nlm.nih.gov/gene/108217739) | 4 | 4977146 | 4966978 | [XP017246108.1](https://www.ncbi.nlm.nih.gov/protein/XP_017246108.1) | 191 | *Dct34200* |
| ^▲^*SOC1* | [108217854](https://www.ncbi.nlm.nih.gov/gene/108217854) | 4 | 5039107 | 5029244 | [XP017246237.1](https://www.ncbi.nlm.nih.gov/protein/XP_017246237.1) | 191 | *Dct34200* |

^a^ The carrot genome sequences were from https://www.ncbi.nlm.nih.gov/genome/?term=carrot (Iorizzo et al. 2016).

^▲^The gene didn’t have the complete domain according to the protein sequence compared with the references.

**Supplementary Table S3**

**The primers for reverse transcription PCR and rea**l-time qPCR analysis

| Use | Gene | Forward primer sequence (5′-3′) | Reverse primer sequence (5′-3′) | Size of the fragments /bp |
| --- | --- | --- | --- | --- |
| Reverse transcription PCR | *DcGI* | CCGTGGTCATCGTCTTTAGC | GCACCCCCTTTTTCATCTTT | 2624 |
|  | *DcCOL2* | CCACAGATTCCCAAACTAAC | GCTGCAGAAGTAGATTATGTCA | 1303 |
|  | *DcCOL4* | CACCATTAAATTGACTGTCCAG | TGACATTGATTTGTTTCATTTTATT | 1505 |
|  | *DcCOL5a* | TACTTGCCCTATTTCAATAC | TACTATTCGTACAAAAACTAGG | 1200 |
|  | *DcCOL5b* | ACCTCTCCAATACAGCAATACAC | ACGCATTTTCATCTTGGTGT | 1412 |
|  | *DcCOL13* | GGGTCCACTGTGCTCAAAAG | GCAAACCTAGTCCCAAAAAGA | 1251 |
|  | *DcCOL15* | TGACATCTCTGCTTTGACTCG | AGGAATACAAGGTCTCCAAAACT | 1665 |
|  | *DcFT1* | AAAGGGAGAAAATAATACAAAG | ATACAATCATACACACGCAAG | 600 |
|  | *DcSOC1b* | TCCTCCACCTAAATAATCTCTTCT | TCCATTCCCATCTACCAACC | 996 |
|  | *DcSOC1c* | CCCTTCCATTTCCATCTCC | ATAAATGTTTGTGACCTCCCTG | 1086 |
| Real-time qPCR | *DcGI* | TAGCATCGTCCATAACCCTG | TTGGCAGCAGTATCAAGAAAT | 144 |
|  | *DcCOL2* | CAGGCTCAGCCGAAACAA | GCCTTTAGAGGGTCTTGGA | 183 |
|  | *DcCOL4* | CTGCTCCTGCTCTTGTTGAT | TCTGTCATGGCATTGTGGTC | 157 |
|  | *DcCOL5a* | TTTGAGCGAGTCTGTATCCG | GCAGTCCCATTGTTAGAGGTAT | 182 |
|  | *DcCOL5b* | AGGAGACTAAGTTGTCAGCGG | AACACTATCAGCACCAGTACCAC | 152 |
|  | *DcCOL13* | TTCAGTCCCTGTTGCCTTAC | ATTTCTTCTGCATCCTCGTC | 139 |
|  | *DcCOL15* | GGTGCCTAGTAGTAGTTTGTTTGG | AATGCTCGTCTCCGTCTTGT | 170 |
|  | *DcFT1* | GACCCCGATGCTCCTACTCCAA | TGAACCAGACTCCCGCTGACAG | 300 |
|  | *DcSOC1b* | AAGAATCGCCAGAAGACGG | CGCTTGAATCTCTTTTCTGGA | 109 |
|  | *DcSOC1c* | GAAAAGGAGAAGACCCTAGCA | GTCCAATGTAGAGTTCAGTTTCC | 148 |
| Reference gene | *Tublin* | GAATACCAGCAGTACCAAGA | CATTACATATCTTGATGAGCC | 88 |
